# The International Congress of Surgeons, 1908

**Published:** 1909-06

**Authors:** Edgar R. McGuire

**Affiliations:** Associate Surgeon, Buffalo General Hospital


### The International Congress of Surgeons, 1908.

By EDGAR R. McGUIRE, M. D.
Associate Surgeon, Buffalo General Hospital.

THE first meeting of the International Congress of Surgeons was held in Brussels, 1905. It is an organisation of representative surgeons from every country, America having about sixty members. Meetings are held every three years. Professor Kocher of Bern, was the first president; Professor Czerny of

Heidelberg, was next, and Professor Lucas-Championiere, Paris, the present one. The executive committee is made up of one member from each country and it forms the governing body. Dr. Roswell Park, of Buffalo, has been the American member of this committee since the organisation of the society.

The second meeting was also held in Brussels, which is in many respects an ideal location for such a meeting. It is centrally located, well equipped with clinical facilities, so essential for such a meeting, and has several buildings admirably adapted for so large a gathering as the Congress brings. The meeting was held in one of the buildings recently erected for the International Exposition, to be held in 1910.

A large part of the building was given to the exhibition of surgical instruments. Almost every important manufacturer in the world had an exhibit, making this a decided feature. There was also a large cancer exhibit where gross specimens of almost every conceivable malignant lesion were shown. These were arranged so that sections could be seen of any specimen.

There was a first class café in the building, so that most of the members remained all day, taking lunch in the building.

The program was divided into five main subjects: (1) symposium on cancer; (2) surgery of the liver and biliary passages; (3) anesthesia; (4) surgery of the spine; (5) hernia.

A glance at the complete program will readily convince one of the interest of such a meeting. Seldom has one the opportunity to see so many men of international reputation in one room, discussing the same subject.

Following is a brief outline of the program:

### TREATMENT OF CARCINOMA.

I. Report on the nature of the cancerous process, by Professor Roswell Park. II. Report on the pathogeny of epithelioma, by Dr. A. Delbet; on the pathogeny of cancer, by Dr. Girard. III. Report on the treatment of cancer of the lips, by Dr. von Bonsdorff. IV. Report on cancer of the mouth and tongue, by Prof. J. Collins Warren. V. Report on treatment of naso-pharyngeal and laryngeal cancer, by Dr. Glück: systematic ablation of the naso-pharynx, by Dr. Durant. VI. Report on the treatment of carcinoma of the esophagus, stomach, liver, biliary ducts, pancreas and peritoneum, by Prof. Czerny. (1) results of twenty gastrectomies for carcinoma of the stomach, by Dr. Derujinski; (2) conditions favorable for excision in cancer of the small curvature of the stomach, by Dr. H. Delagenière; (3) surgical treatment of carcinoma of the liver, by Dr. P. Peugniez; (4) carcinoma of the pancreas, by Dr. Verhoef; (5) primary sarcoma of the spleen, by Dr. d'Arcy Power. VII. Report on the treatment of cancer of the intestine, by Prof. Voelcker; Carcinoma of the rectum with early vesical troubles, by Dr. Ch. Girard. VIII. Report on the treatment of uterine cancer, by Dr. J. L. Faure: (1) carcinoma of female genital organs; carcinoma of the uterus, by Dr. Delétrez; (2) carcinoma of female genital organs. Uterine cancers, by Dr. Jacobs; (3) end-results of operations for uterine cancer, by Dr. Wertheim. IX. Report on the treatment of cancer of the genitourinary organs, by Dr. Legueu. X. Report on the treatment of epithelioma of the skin, by Dr. Morestin. XI. Report on the treatment of carcinoma of the breast, by Prof. A. Depage. (1) carcinoma of the breast by Dr. Delétrez; (2) carcinoma of the breast, by Dr. Steinthal; (3) treatment of carcinoma of the breast, by Dr. Vanverts. XII. Report on carcinoma of the renal capsule, by Dr. Tavel. XIII. Report on the treatment of cancer by radio- and radium-therapy, by Prof. Sequeira; on the value of fulguration in the treatment of carcinoma, by Prof. H. Reynes. XIV. Report on the treatment of inoperable cancer, by Henry Morris: (1) treatment of inoperable uterine carcinoma by Dr. Delétrez; (2) palliative treatment of inoperable cancer, by Dr. Lovell Drage. XV. Report on end-results of surgical treatment of cancer, by Prof. Dollinger; operative results in carcinoma, by Dr. Korteweg.

### SURGERY OF THE LIVER AND BILIARY DUCTS.

I. Report on lithiasis, by Prof. Kehr: (1) usefulness of the gradual section of the ducts in operations for lithiasis; its indications, by Dr. H. Delagenière; (2) on the surgery of the biliary ducts, by Dr. J. Bakès; (3) on the perforation of the gall-bladder, by Dr. Max Sträter; (4) on gallstones, by Dr. Steinthal. II. Report on surgical treatment of cirrhoses of the liver, by Dr. Koch; surgical treatment of the cirrhosis of the liver, by Dr. P. Peugniez. III. Report on treatment of angiocholitis, by Prof. Quénu and Dr. Duval. IV. Report on treatment of abscesses of the liver, by Dr. Legrand. V. Report on abscesses of the liver, by Dr. Voronoff. VI. Report on treatment of tumors of the liver, by Prof. Payr.

### ANESTHESIA.

I. Report on general anesthesia, by Prof. Vallas: (1) on the mixed-narcosis with ethylchlorid and oxygen, by Dr. G. Lotheissen; (2) technics and indications of the ether-narcosis, by Prof. J. L. Reverdin and Ch. J. Bergalonne; (3) on mixed-narcosis with scopolamin-morphin and chloroform, by Drs. Rouffart and Walravens. II. Report on medullary anesthesia, by Prof. Rehn; results of 1,650 cases of medullary anesthesia, by Dr. Fr. Zahradnicky. III. Report on local anesthesia, by Dr. Mac

Arthur: (1) local anesthesia, by Dr. F. Moty; (2) endoneural anesthesia of the limbs, by Dr. Alexius von Hintz.

SURGERY OF THE SPINE.

I. Report on traumatisms of the spine, by Prof. de Quervain. II. Report on tumors of the spine, by Prof. Berard; tumor of spinal cord.—Operation.—Radical cure, by Dr. McCosh.

HERNIA.

I. Report on etiology of hernia, by Prof. Forgue; etiology of hernia, by Dr. William Sheen. II. Report on inguinal hernia, by Dr. Alessandri: (1) the inguinal hernias in adults, by Dr. F. Moty; (2) on 7,000 operations for inguinal hernia, by Dr. Petrovitch; (3) treatment of the sac in Bassini's operation, by Dr. V. Subbotitch; (4) operative procedures for inguinal hernia, by Dr. J. Vanverts. III. Report on crural hernia, by Dr. Hildebrand: (1) operation for crural hernia, by Dr. G. Lotheissen; (2) radical cure of crural hernia by the procedure of the "double curtain" (procédé du double rideau), by Dr. Léon Bérard; (3) treatment of crural hernia, by Dr. J. Vanverts. IV. report on umbilical hernia, by Prof. Fraenkel. V. Report on hernia in children, by Dr. J. Lorthioir; early interference for umbilical congenital embryonic hernia, by Dr. Nove-Josserand. VI. Report on operative procedures and end-results of the radical cure of hernia, by Prof. Kalliontzis; operative procedures for treatment of hernia, by Dr. Verhoef.

Abstracts of each paper, limited to fifteen minutes each were read. The entire papers are to appear later in the published transactions of the society. The discussions were very valuable, as every one seemed to express himself perfectly freely. This feature was illustrated in Mr. Moynihan's discussion of Professor Kehr's paper on gallstone disease. Mr. Moynihan said it was seldom his privilege to hear and see such an array of material and figures as Kehr had presented and still be compelled to disagree in almost every particular. This discussion especially pleased the American members, as Mr. Moynihan's advanced position was in complete accord with their views. He maintained in part that gallstones always caused symptoms if we could but recognise them; that medical treatment might relieve symptoms but failed to influence the pathology of the disease; that if we were to avoid the experience of Kehr, who confessed he was compelled to operate most of his cases in the late stages of contracted gallbladder, duct stone, jaundice, pancreatic disease, and the like, we must recognise the condition early and then proceed with surgical relief.

The social features formed a very delightful part of the meeting. Formal dinners were given every evening, usually more than one each evening, where one might see from fifty to one hundred guests at table in a private home. Without proper attention to details, I can imagine that such a dinner might prove very monotonous. If, for instance, one were limited to English in his conversation and one's immediate neighbors at the dinner spoke Russian on one hand and perhaps Italian on the other, conversation might somewhat resemble that of deaf mutes.

One of the most valuable features of such a congress consists in meeting so many famous men together. To see and hear them discuss papers gives one a better acquaintance than years of reading could possibly do.

There seemed to be a very earnest desire to have the next meeting in America. It was thought wise, however, on account of the youth of the organisation, to remain in Europe, and Brussels was again chosen as the meeting place for 1911.

Our trip gave us an opportunity of seeing at close range the condition of the average American student in Europe.

There is prevalent in America an entirely erroneous idea as to the relative merits of European and American surgery. From the time I began the study of medicine I heard of the wonders of the old world in matters pertaining to medicine and surgery. Some of my friends returning from study in Europe brought back wondrous tales of foreign surgery. It is high time to reverse such opinions, for the best surgery in the world today is done in America.

There are many places in Europe where one may see most excellent work, and a visit to such places is well worth while; but for the recent graduate to go to Europe to study surgery is exceedingly unwise.

It is really a pitiable sight to see fifty or more young Americans in Berlin struggling with the difficulties of a foreign language, taking courses in surgery, trying to see work here and there, when at home they could see better work and just as much of it with infinitely less inconvenience and expense.

The value of a European trip is inestimable. The refining influence of travel, the historic scenes, the beautiful galleries, the insight into the life of each of the great cities, the acquirement of languages, all constitute advantages not to be had at home. But let us have a true estimate of the surgery of our own country. When Europeans fully realise the high character of the surgical work done in America, travel will be in the other direction. The place to study surgery today is in America.

430 Delaware Avenue.

